# The Impact of Gestational Diabetes on Kidney Development: is There an Epigenetic Link?

**DOI:** 10.1007/s11892-024-01569-9

**Published:** 2024-12-18

**Authors:** Giovane G Tortelote

**Affiliations:** Section of Pediatric Nephrology, Department of Pediatrics, Tulane University School of Medicine, New Orleans, LA 70112 USA

**Keywords:** Gestational diabetes, Kidney development, Epigenetics, CAKUT, Oligonephropathy, Fetal reprogramming

## Abstract

**Purpose of Review:**

This review explores the mechanisms through which gestational diabetes mellitus GDM impacts fetal kidney development, focusing on epigenetic alterations as mediators of these effects. We examine the influence of GDM on nephrogenesis and kidney maturation, exploring how hyperglycemia-induced intrauterine stress can reduce nephron endowment and compromise renal function *via* dysregulation of normal epigenetic mechanisms.

**Recent Findings:**

In addition to metabolic impacts, emerging evidence suggests that GDM exerts its influence through epigenetic modifications, including DNA methylation, histone modifications, and non-coding RNA expression, which disrupt gene expression patterns critical for kidney development. Recently, specific epigenetic modifications observed in offspring exposed to GDM were implicated in aberrant activation or repression of genes essential for kidney development. Key pathways influenced by these epigenetic changes, such as oxidative stress response, inflammatory regulation, and metabolic pathways, are discussed to illustrate the broad molecular impact of GDM on renal development. Finally, we consider potential intervention strategies that could mitigate the adverse effects of GDM on kidney development. These include optimizing maternal glycemic control, dietary modifications, dietary supplementation, and pharmacological agents targeting epigenetic pathways.

**Summary:**

Through a comprehensive synthesis of current research, this review underscores the importance of early preventive strategies to reduce the burden of kidney disease in individuals exposed to GDM and highlights key epigenetic mechanisms altered during GDM that impact kidney development.

## Introduction

According to the Department of Health and Human Services, obesity is a significant public health issue affecting roughly 40% of women of reproductive age [1]. Maternal obesity is a modifiable risk factor known to contribute to adverse pregnancy outcomes. It carries substantial short-term and long-term health consequences, negatively impacting a child’s development into adulthood [2–6].

Gestational maternal obesity has been associated with both low birth weight (birth weight less than 2,500 g) and macrosomia (birth weight greater than 4,000 g). Babies born under these conditions have a 50% higher risk of developing childhood obesity and a 35% higher risk of premature cardiovascular death [5, 7, 8].

Mechanistically, obesity leads to changes in the uterine environment that alter placenta function and embryonic development. Obesity leads to abnormal hormonal responses during gestation, such as increased levels of leptin, insulin, and insulin-like growth factor (IGF), which influence fetal development. Additionally, obesity may affect nutrient availability, altering metabolic signals and the expression of genes critical for proper embryonic development. Finally, obesity is associated with increased levels of inflammatory cytokines and oxidative stress markers, which can disrupt normal embryonic development. Notably, while maternal obesity affects the development of the entire embryo, certain tissues—such as the embryonic kidneys—are particularly vulnerable to alterations during pregnancy [6, 8–12].

Obesity is a major risk factor for the development of gestational diabetes mellitus (GDM) [13]. The likelihood of developing GDM is two to three times higher in women with obesity compared to those with a healthy weight. GDM exposes the developing fetus to altered insulin secretion and sensitivity, resulting in high blood glucose levels, which disrupt normal fetal metabolism and development and, in particular, kidney development [14–16]. GDM is a frequent pregnancy complication (on average 14%, range 1 to 25%) with significant implications for both maternal and fetal health [17]. The variability in the global prevalence of GDM appears to be rooted in the reported regional differences. Southeast Asia, the Middle East, and North Africa report the highest GDM prevalence, while regions such as the Americas, Africa, and the Western Pacific show moderate prevalence levels. Europe, in contrast, has the lowest GDM rates among World Health Organization regions [17–21]. These global differences may partly arise from varying diagnostic criteria across countries. However, they also likely reflect significant disparities in GDM risk among different racial and ethnic communities, underscoring the need for culturally tailored health interventions or more personalized medicine.

## Impact of Maternal Metabolic Adaptations, Obesity and GDM on Pregnancy Outcomes and Offspring Health

Pregnancy is the physiological condition in which a fertilized egg develops into a fetus inside a woman’s uterus. It begins with the implantation of the embryo into the uterine lining and typically lasts around 40 weeks, divided into three trimesters. Pregnancy leads to complex physiological, hormonal, and metabolic changes in the mother to support fetal growth, development, and preparation for childbirth. It concludes with the delivery of the baby through vaginal birth or cesarean section. Over the course of a healthy pregnancy, maternal physiology undergoes significant adaptations to support fetal growth, including critical changes in insulin sensitivity tailored to different pregnancy stages. In early pregnancy, maternal insulin sensitivity increases, promoting glucose uptake into adipose tissue and preparing for the increased energy demands of later pregnancy stages. As pregnancy progresses, however, maternal metabolism shifts to a state of relative insulin resistance. This transition results in modestly elevated maternal blood glucose levels, which are readily transported across the placenta to support fetal growth. Additionally, insulin resistance stimulates endogenous glucose production and mobilizes fat stores, leading to higher levels of blood glucose and free fatty acids [22–25].

To maintain proper glucose regulation, maternal pancreatic β-cells increase insulin secretion to compensate for the reduced tissue sensitivity to insulin. In cases of GDM, pancreatic β-cells fail to adequately offset the increased insulin resistance, leading to maternal glucose intolerance and the onset of GDM. Obesity exacerbates this process by inducing insulin resistance and hyperinsulinemia, which are thought to be driven by low-grade systemic inflammation and subclinical endotoxemia [6, 8, 22, 24].

Obesity during pregnancy significantly impacts glucose metabolism, causing impaired fasting glucose regulation in early pregnancy and a pronounced increase in peripheral and hepatic insulin resistance. Consequently, pre-pregnancy obesity-related insulin resistance greatly increases the risk of developing GDM. Changes in maternal insulin sensitivity throughout pregnancy are partly influenced by fat mass, which increases in both normal-weight and obese women during pregnancy. However, obesity-related insulin resistance reduces the effect of insulin on lipolysis, altering lipid metabolism and leading to a several-fold increase in triglyceride and cholesterol levels late in pregnancy [6, 8, 26].

Almost all obese women display some degree of dyslipidemia throughout all stages of pregnancy [5, 8]. This suggests that fetuses in obese pregnancies are exposed to high levels of free fatty acids at every stage of development. This chronic exposure to free fatty acids can exert a lipotoxic effect, contributing to inflammation and endothelial dysfunction. These disruptions lead to altered placental metabolism and function, increasing the supply of excess lipids and glucose to the fetus [24, 25, 27].

The combination of nutrient excess, hormonal imbalances, and placental dysfunction creates an adverse *in-utero* environment that may increase the risk of metabolic diseases in offspring. These mechanisms underscore the importance of managing maternal obesity and metabolic health during pregnancy to reduce risks to both the mother and child.

The developing kidney is susceptible to environmental influences during development. Alterations in kidney development due to obesity and GDM can reduce nephron endowment, impair renal function, and increase susceptibility to hypertension, which affects 35% of the world’s population, and kidney disease, which impacts roughly 12% of the world’s population [16, 28–33]. Studies have suggested that the offspring of mothers with GDM may have a higher risk of developing congenital anomalies of the kidneys and urinary tract (CAKUT) [31, 32, 34–36]. While research indicates a potential link, the exact evidence of the direct impact of gestational diabetes on kidney development in the offspring is still considered limited and requires further investigation [37, 38].

GDM is thought to impact kidney development through both direct metabolic effects and epigenetic modifications. Epigenetic changes, such as DNA methylation, histone modifications, and microRNA expression, are crucial during fetal development and can respond dynamically to maternal hyperglycemia [39–43]. In the context of GDM, these changes may modify gene expression and disrupt developmental pathways, potentially predisposing offspring to long-term cardio-renal-metabolic disorders later in life [31, 43, 44].

This review aims to explore the influence of GDM on fetal kidney development. We will summarize current knowledge on how GDM affects renal development and highlight how epigenetic alterations may contribute to the risk of kidney disease in adulthood.

## Kidney Development at a Glance

**Fig. 1:**
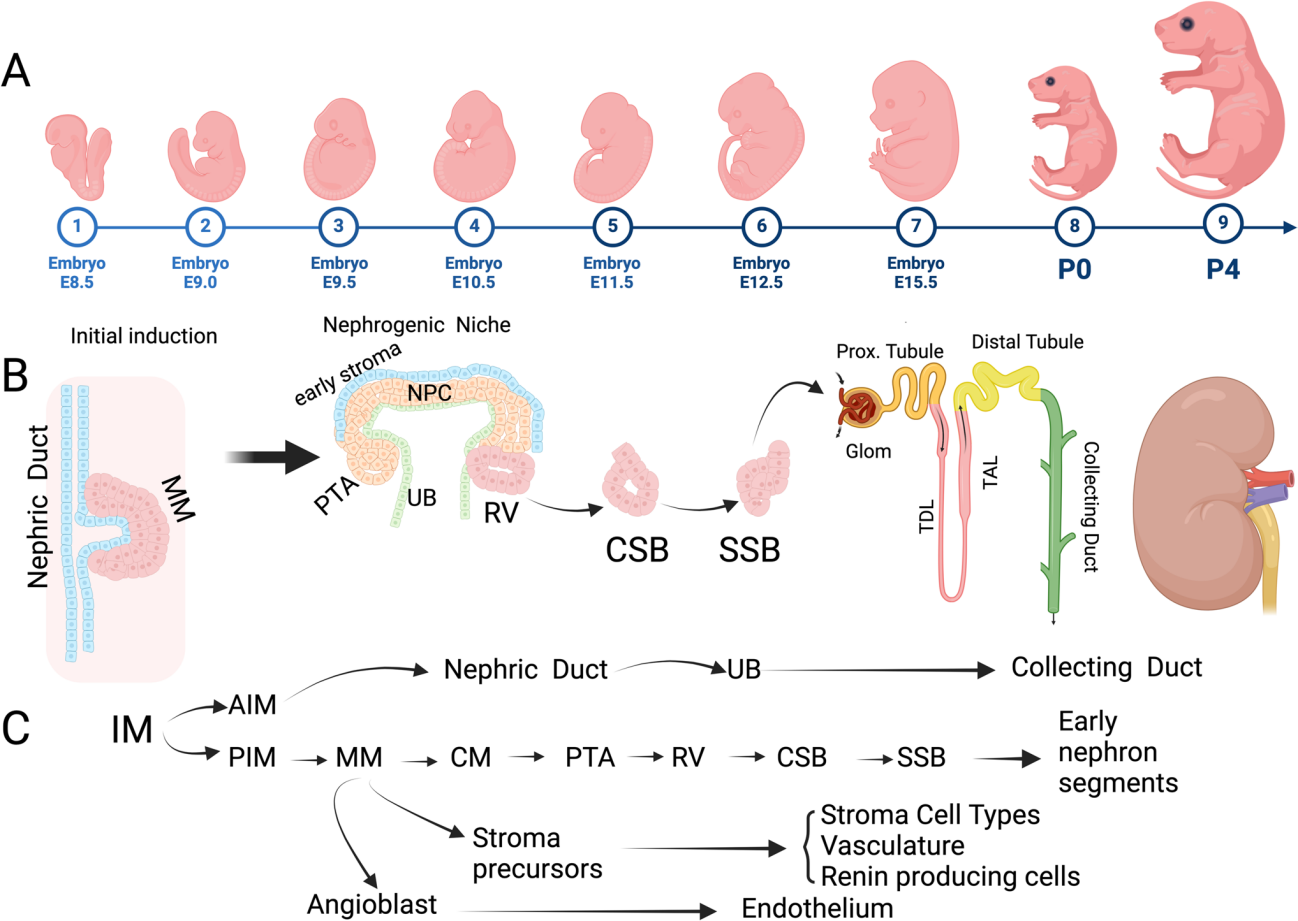
The dynamic and coordinated process of kidney development from early progenitors to the formation of functional nephrons. Timeline of embryonic kidney development from embryonic day 8.5 (E8.5) to postnatal day 4 (P4), showing progressive stages of fetal development (**A**). Schematic representation of nephrogenesis within the embryonic kidney. the nephric duct is induced by the adjacent metanephric mesenchyme (MM) and invades the MM to form the nascent UB. Next, the reciprocal inductive processes continue to form the nephrogenic niche. The nephrogenic niche contains early stromal cells, nephron progenitor cells (NPC), and the ureteric bud (UB). Nephron formation progresses from pretubular aggregates (PTA) to renal vesicles (RV), and subsequently to comma-shaped bodies (CSB) and S-shaped bodies (SSB), which differentiate into early nephron segments, including proximal tubules, glomeruli (Glom), thick ascending limbs (TAL), thin descending limbs (TDL), distal tubules, and collecting ducts (**B**). these developmental processes repeat themselves in a modular and hierarchal manner until the kidneys are formed. The tissue ontogeny of kidney development starts with the Intermediate mesoderm (IM), which gives rise to anterior (AIM) and posterior intermediate mesoderm (PIM), which differentiate into metanephric mesenchyme (MM). MM progresses through cap mesenchyme (CM), pretubular aggregate (PTA), renal vesicle (RV), and later stages of nephron development. Stroma precursors give rise to stromal cell types, vasculature, renin-producing cells, and endothelia. The nephric duct forms the ureteric bud (UB), which later gives rise to the collecting duct system (**C**). Created with Biorender.

Kidney development, or nephrogenesis, is a complex, multistage process that begins in early embryonic life and continues until shortly after birth in some mammals. In humans, kidney development begins around gestational week 5 and is mostly complete by week 35 of the 40-week gestation period. In contrast, in mice, kidney development starts at approximately day 10 of gestation and continues postnatally, ceasing around day 5 after birth, as their gestation lasts about 20 days (Fig. 1A) [45–50].

The kidneys are two bean-shaped organs located in the retroperitoneal cavity, with most of their mass slightly below T12 vertebrate. The kidney performs essential functions in the body, including the elimination of toxic wastes such as urea and creatinine, and the reabsorption of essential molecules such as proteins, sugars, and micronutrients. Additionally, the kidneys regulate blood volume, blood pressure, osmolarity, and pH balance [51].

The kidneys originate from the intermediate mesoderm, which forms the pronephros, mesonephros, and metanephros in a sequential process. The metanephroi are ultimately the functional mature kidneys. Reciprocal signaling from the ureteric bud (UB), an outgrowth from the mesonephric duct, and the adjacent mesenchymal cells (metanephric mesenchyme), mark the beginning of nephrogenesis (Fig. 1B) [46]. Three progenitor lineages emerge during nephrogenesis: the nephron progenitor cells (NPC) lineage. These cells are characterized by the expression of *Six2*,* Cited1*,* Osr1*,* Sall1* and several other NPC identity genes. The stroma progenitor’s lineage originally shows *Foxd1*,* Lgals*,* Dcn* and the expression of several other markers. The ureteric bud lineage is characterized by the expression of *Ret*,* Wnt11*,* Wnt9b*, and *Gfra1*. Further, the interaction between the NPC and cells from the other two lineages coordinates normal kidney development [45, 46]. The UB undergoes branching morphogenesis, forming a tree-like structure that will become the renal collecting system. NPCs differentiate into specialized cell types, forming nephrons—the functional units of the kidney—through a series of stages (Fig. 1B, C). NPCs proliferation and differentiation are governed by a complex network of cell types and molecules, for instance, the branching UB tips secrete growth factors such as GDNF and Wnt proteins that influence NPC fate decisions [45, 46, 52]. Kidney development is a modular process that includes the formation of transitory structures such as the pretubular aggregate, the renal vesicle, the comma-shaped body, and the S-shaped body. These transitory structures further develop into functional nephrons, that comprise the glomeruli, proximal and distal tubules, and the loop of Henle (Fig. 1C). Key signaling pathways (e.g., Wnt, Notch, BMP, GDNF etc.) guide the patterning, segmentation, and cell fate within nephrons. The stroma progenitors give rise to all stroma-derived cells, the support cells in the mature kidney, the vasculature, and the mesangium [53]. Nephron formation completes before birth in some mammals, such as humans, and after birth in others, like mice, with the number of nephrons determined by a balance between NPC proliferation and differentiation rates [45, 46].

Disruptions in these processes can lead to conditions like oligonephropathy (low nephron endowment) at birth and congenital anomalies of the kidney and urinary tract (CAKUT). Throughout development, a balance of self-renewal and differentiation of NPCs is required to sustain nephron formation. NPCs are gradually depleted as nephrogenesis progresses, eventually ceasing with the depletion of this progenitor Pool at around 35 weeks of gestation in humans and roughly postnatal day 5 in mice [32, 50, 54–56].

The newly formed nephrons mature and become vascularized, establishing connections with the glomerular capillaries. This vascular integration is crucial for kidney filtration function, enabling the transition to fully functional kidneys postnatally. After birth, kidney size and nephron complexity increase as the organ adapts to the needs of the organism, with nephron enlargement and increased tubule length [45, 50, 57, 58]. Genetic mutations or adverse environmental factors (e.g., poor diet during gestation, gestational diabetes, drug use, etc.), can affect nephron number and function, contributing to kidney-related diseases later in life [51]. Importantly, after the developmental periods mentioned earlier, the kidneys no longer retain a pool of progenitor cells. This means that any nephrons lost cannot be regenerated. Therefore, acquiring and maintaining an adequate number of functional nephrons is crucial for preventing hypertension and chronic kidney disease (CKD) later in life.

### Developmental Origin of Hypertension and Chronic Kidney Disease

Identifying the mechanisms regulating mammalian kidney development is essential for understanding chronic kidney disease (CKD) etiology. In the U.S., around 30 million adults (15% of the population) have CKD, with hypertension—a major CKD risk factor—impacting roughly 35% of adults [33, 59].

In the 1980s and 1990s, the research of Drs. Barker and Brenner played a foundational role in understanding the developmental origins of kidney disease and have significantly influenced the field of nephrology and developmental programming.

Barker’s research in the 1980s focused on epidemiological studies linking low birth weight to increased risks of chronic diseases in adulthood, such as cardiovascular disease, hypertension, and type 2 diabetes. His hypothesis proposed that poor nutrition or other environmental factors during pregnancy could “program” the fetus, leading to permanent changes in structure, function, and metabolism that predispose individuals to diseases later in life [60].

Although Barker’s work primarily focused on cardiovascular disease, it became clear that kidney development could also be affected by adverse intrauterine environments. The concept was expanded to suggest that reduced nephron endowment might be an important developmental factor leading to CKD and hypertension in adults. This hypothesis provided a developmental framework for understanding the developmental origins of kidney disease [60–62].

Later, Dr. Brenner proposed that individuals with a reduced number of nephrons (whether due to genetic, environmental, or developmental factors) are more prone to kidney disease. Brenner’s research highlighted that individuals with fewer nephrons had to compensate by hyperfiltration (increased workload per nephron), which over time led to glomerular hypertension, progressive glomerular damage, and chronic kidney disease [63–68].

Brenner’s hypothesis agreed well with Barker’s, as a low nephron number could result from a poor fetal environment. Brenner’s work solidified the link between impaired nephrogenesis and later-life susceptibility to hypertension and CKD. The understanding of nephron endowment as a critical factor shaped research into prenatal influences on kidney development, reinforcing the developmental origins of kidney disease. Together, the work of Barker and Brenner led to a broader understanding that kidney disease could originate *in utero*, influenced by maternal diet, stress, and other factors during pregnancy. This has driven research into interventions that might improve maternal-fetal health to prevent CKD later in life [64, 65, 67, 69, 70].

Nephron endowment at birth is critical for long-term renal and cardiovascular health and is contingent on the NPC pool [62, 66, 67, 71]. During development, NPCs face variable environmental conditions influenced by, maternal diet, the mother’s pathological state, pollutants, and intrinsic factors such as their location and spatial interactions within the nephrogenic niche [72–75]. Challenges such as gestational diabetes, premature birth, placental abnormalities, or nutritional deficiencies can impose metabolic stress, impacting kidney development [31, 32, 51, 54, 56, 68, 76–78]. To overcome environmental changes, NPCs must adapt to the evolving milieu surrounding them. Recently, we showed that glucose-derived acetyl-CoA is a key factor in maintaining the NPC pool. Interestingly, acetyl-CoA is a major substrate for epigenetic regulation in cells [79, 80].

Previous research, including ours, has demonstrated that an imbalanced diet lacking either macronutrients (Protein, carbohydrates) or even micronutrients (vitamin A, E, and Iron) and disruption of cell metabolism during gestation can negatively impact normal cell metabolism and kidney development [51, 74, 81–88]. Unfortunately, not only diet [51, 56] and prematurity [89–91] but also metabolic diseases such as gestational diabetes [92, 93] can lead to oligonephropathy, which increases the risk of CKD later in life [63, 78]. Children born under these conditions have few, sometimes no, therapeutic options. Thus, developing new therapeutic strategies to improve embryonic development and foster premature baby development is of utmost clinical relevance.

## Impact of GDM on Fetal Kidney Development

**Fig. 2:**
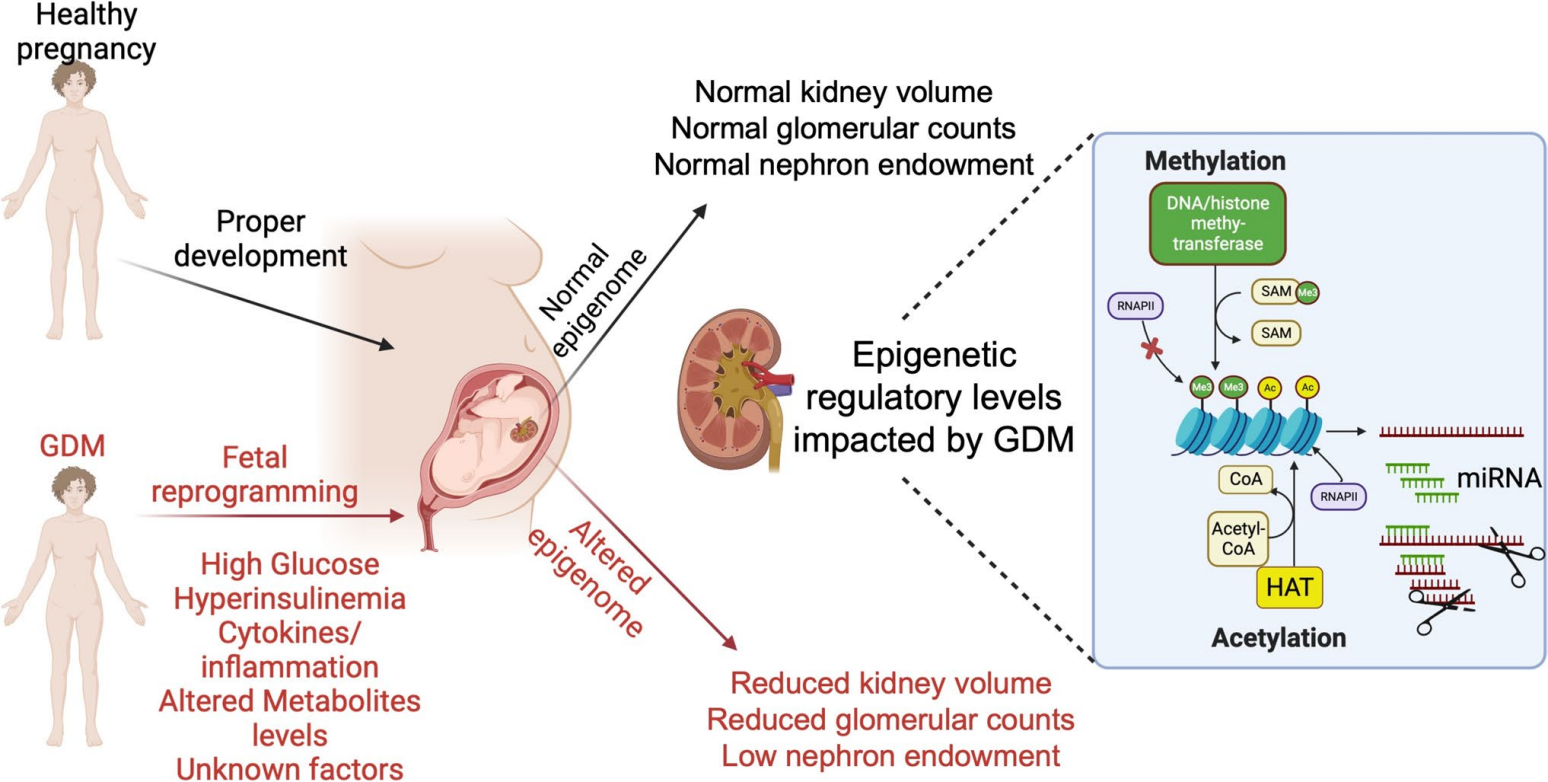
Gestational diabetes mellitus alters fetal epigenetic programming, impacting nephrogenesis. In GDM, elevated levels of glucose, hyperinsulinemia, inflammatory cytokines, and altered metabolite levels result in fetal reprogramming. This leads to epigenomic changes in the developing kidney, specifically in DNA and histone methylation and acetylation. These epigenetic modifications include DNA/histone methylation via methyltransferases and histone acetylation through histone acetyltransferases (HATs), which affect transcriptional activity and microRNA (miRNA) expression. The altered epigenome results in reduced kidney volume, decreased glomerular counts, and lower nephron endowment, contributing to increased risk for chronic kidney disease in offspring. Abbreviations: GDM, gestational diabetes mellitus; HAT, histone acetyltransferase; miRNA, microRNA; SAM, S-adenosyl methionine; RNAPII, RNA polymerase II. Created with Biorender.

The incapacity of maintaining blood pressure control is a sign of kidney malfunction. Experimental models show that maternal hyperglycemia is linked to several adverse renal outcomes in offspring, including a reduced nephron count, elevated blood pressure, microalbuminuria, and decreased glomerular filtration rate (Fig. 2) [32-94]. In humans, GDM descendants show higher mean body mass index and systolic and diastolic blood pressure compared to non-recorded-GDM descendants at similar ages [95]. Furthermore, individuals from mothers who had diabetes exhibit reduced renal function compared to those with diabetic fathers, suggesting that lower nephron numbers may result from exposure to *in-utero* gestational diabetes [96]. Additionally, maternal diabetes is associated with a threefold increase in the risk of renal abnormalities, such as renal agenesis and dysgenesis [97]. These findings highlight the long-term renal impact of GDM on offspring.

A recent study compared 42 children born to diabetic mothers with 21 children of non-diabetic mothers. The findings revealed that children of diabetic mothers had significantly reduced renal cortex volumes and higher albumin excretion levels compared to the controls, likely due to a reduced nephron count [98]. Conversely, a study by Aisa et al. (2019) found that neonates of diabetic mothers who maintained strict normoglycemic control showed no differences in kidney volume compared to controls. However, the offspring of mothers with poor glycemic control had significantly lower renal volumes [36], a key risk factor for developing CKD later in life.

Mechanistically, GDM exposes the developing fetus to elevated glucose levels, which significantly impacts various aspects of kidney development [34, 35, 38, 92, 93]. Fetal kidneys are highly sensitive to environmental stressors due to the complex and timed process of nephrogenesis. Exposure to GDM can impair nephrogenesis, leading to a reduced nephron endowment, which is associated with a higher risk of kidney disease, hypertension, and metabolic disorders later in life (Fig. 2) [36, 38, 92, 93, 99–101]. Studies with rodents have shown that GDM-associated hyperglycemia disrupts critical processes in kidney development, including NPC’s proliferation, differentiation, and apoptosis [92, 93]. Notably, high glucose levels may induce oxidative stress in NPCs, causing cellular damage and premature exhaustion of these progenitors. This exhaustion leads to oligonephropathy, which compromises kidney function and increases the risk of hypertension and CKD later in life [34, 35, 38]. GDM may potentially alter levels of growth factors like insulin and IGF-1, both of which are critical for proper kidney development and cell metabolism regulation [102].

A recent study found that a diabetic intrauterine environment hinders the differentiation of NPC into nephrons, possibly due to its impact on signaling pathways involved in proper kidney development, such as Notch and Wnt/β-catenin [103]. GDM-exposed offspring of rodents exhibit smaller kidneys with fewer nephrons, and increased expression of inflammatory cytokines and markers of fibrosis in the kidney tissues [104]. In another study with rodents, GDM resulted in a predisposition to high-salt dietary-induced vascular dysfunction and inflammation later in life [105]. Such findings suggest that GDM exposure induces a pro-inflammatory renal environment, which predisposes the offspring to renal dysfunction. In line with these findings, two recent studies showed that maternal diabetes dysregulates signaling pathways that are required for proper kidney development [92, 93]. Therefore, the effects of GDM on healthy kidney development involve multiple molecular mechanisms, complicating the design of effective therapies to prevent abnormal kidney development caused by GDM.

## Epigenetics, Cell Metabolism, and Kidney Development

Epigenetics refers to heritable changes in gene expression that do not involve alterations in the DNA sequence itself. Cell metabolism plays a key role in linking epigenetic regulation to nephron progenitor cell fate decisions. Epigenetic regulation modifies how genes are expressed by altering the chromatin structure (the complex of DNA and proteins in the nucleus) and by adding or removing chemical modifications to DNA and histones (the proteins around which DNA is wrapped). These modifications include DNA methylation, histone modifications (methylation, acetylation, phosphorylation, and other post-translational modifications), and interactions with non-coding RNA, which together regulate gene expression in response to environmental, developmental, and cellular signals [31, 106].

Epigenetic changes are crucial in development because they link response to environmental cues to cellular behavior, such as fate decisions. Although epigenetic changes can be reversible, scientific evidence shows they may play a role in diseases like cancer [107–109], metabolic conditions, and gestational diabetes [43, 110]. During embryonic development, the fetus is especially sensitive to the conditions of the intrauterine environment, and maternal habits or existing pathologies such as GDM can affect normal cell metabolism and both maternal and fetal gene transcription. The reason for that is that cell metabolites are either intermediate or final byproducts of cellular metabolism, which is influenced by dietary habits and physiological and pathological state [111–113]. Cell metabolites have been linked to epigenetic regulation of gene expression [107, 108, 114–117]. They act as substrates for enzymes that facilitate epigenetic modifications [117, 118]. These modifications influence chromatin accessibility and gene expression within cells. As a result, the availability of metabolites provides cells with a direct means to react to environmental signals, by controlling gene expression and cell behavior [104, 108].

In terms of chromatin accessibility regulation, the two major metabolites studied are S-adenosylmethionine (SAM) and acetyl-CoA, which are the two primary substrates for epigenetic modifications that affect gene transcription via post-translational modifications of histone proteins and DNA. While SAM serves as a methyl donor, acetyl-CoA serves as the substrate for the acetylation of histone proteins [108, 119]. However, in addition to cell metabolites, miRNAs are an important layer of gene transcription regulation [120–122].

*SAM* is synthesized in cells primarily through a reaction catalyzed by the enzyme methionine adenosyltransferase (MAT). The process relies on methionine and ATP. Methionine is obtained from dietary sources or recycled within the cell through the methionine cycle. Since it’s an essential amino acid, cells rely on external sources to replenish it regularly. Inside the cell, methionine combines with ATP in a reaction catalyzed by MAT, forming SAM and releasing triphosphate (PPPi). Additionally, the availability of methionine can depend on cellular folate and vitamin B12 levels, which are required for the conversion of homocysteine (a methionine derivative) back into methionine through the methionine cycle. This remethylation process is crucial in tissues where methionine may be low, ensuring an adequate supply for SAM production [123, 124]. Of note, recent work found that methionine supplementation could restore nephron endowment in the offspring of mice fed a hypocaloric diet [82].

Once formed, SAM can donate its methyl group in various reactions, most notably in DNA, RNA, protein, and lipid methylation. This methyl donation converts SAM into S-adenosylhomocysteine (SAH), which can then be broken down into homocysteine, feeding back into the methionine cycle. SAM is an essential methyl donor involved in DNA methylation, histone modification, and polyamine synthesis. Because folate and vitamin B12, play a critical role in SAM production and DNA methylation. Deficiencies in these compounds due to maternal nutrient restriction can lead to altered gene expression and abnormal kidney development. However, folate supplementation was shown to partially restore global methylation and kidney development [124, 125]. SAM is the primary methyl donor for enzymes like DNA methyltransferases (DNMTs) and histone methyltransferases (HMTs), which add methyl groups to DNA and histones, respectively, thereby regulating chromatin structure and gene activity. In NPCs, precise epigenetic regulation is vital for cell identity, differentiation, and proper nephron development. Disruptions in SAM-mediated methylation can lead to abnormal gene expression and impaired kidney development [124, 126].

In 2019, Wanner et al., using a rat model of intrauterine growth restriction demonstrated that postnatal day 1 pups had kidneys with reduced weight and significant DNA hypomethylation compared to controls. This phenotype was replicated by deleting the Dnmt1 gene, which encodes maintenance DNA methyltransferase 1, in NPC. Mechanistically, the resulting decreased nephron count at birth was linked to the lower expression of essential nephrogenesis genes [127]. These findings highlight DNA methylation’s role in linking altered maternal nutrition to renal programming.

Liu et al. (2020) showed that the polycomb proteins EZH1 and EZH2, histone methyltransferases, which add repressive histone modifications (e.g., K4me3+/K27me3+) are required for maintaining NPC populations, and in their ablation, mainly EZH1, leads to depletion of NPCs [128], highlighting the importance of methylation processes to nephrogenesis.

SAM is also a precursor for polyamine synthesis, essential organic cations that support cell proliferation, differentiation, and survival, particularly in rapidly dividing cells like NPCs [129–131]. SAM contributes to glutathione synthesis, a key antioxidant protecting cells from oxidative stress, which can otherwise damage DNA and proteins, impairing cellular processes like proliferation and differentiation [129]. Beyond DNA and histone methylation, SAM is the substrate for the methylation of RNA and proteins, influencing RNA stability, translation, and protein function, all crucial for NPC development and functionality.

*Acetyl-CoA* is a metabolite central to energy production, anaplerotic reactions, lipid biosynthesis, and regulatory acetylation reactions in the cytoplasm and nucleus [132]. In NPCs, glycolysis is the main source of acetyl-CoA, and its inhibition depletes cap mesenchyme, leading to increased NPC differentiation [74]. Outside the mitochondria, in the cytosol and nucleus, ATP-citrate lyase (ACLY) breaks down the mitochondria-derived citrate to produce acetyl-CoA. ACLY reaction is the major source of cytosolic and nuclear acetyl-CoA formation [132]. Genetic ablation of Acly in NPC results in cap depletion, ectopic Wnt4 activation, and reduced nephron numbers at birth. However, supplementing with sodium acetate, which produces acetyl-CoA independently of ACLY, prevented cap depletion in *Acly* mutant kidneys [133]. Therefore, it is likely that acetyl-CoA is regulating NPC fate decisions by changing chromatin accessibility.

Equally important to acetylation is the control of the deacetylation reactions, which are mediated by Histone deacetylases (HDACs). HDAC can deacetylate histone tails and close accessible chromatin, thus negatively modulating transcription. In mice, NPC-specific deletion of *Hdac1* and *Hdac2* led to early postnatal death due to renal hypodysplasia and loss of NPCs. Mechanistically, *Hdac1/2* genetically interacts with regulators of NPC self-renewal such as *Six2*,* Osr1*, and *Sall1*. Hdac1/2 can bind to *Six2* enhancer, impacting its expression and NPC renewal. While mutant NPCs can form renal vesicles, Hdac1/2 mutant kidneys lack nephrons or mature glomeruli. The transcription of several genes, such as *Lhx1*, *Dll1*, and *Hnf1a/4a*, normally expressed in the renal vesicle, was disrupted by the ablation of *Hdac1/2*. Therefore, the epigenetic regulators *Hdac1/2* via histone deacetylation reactions impact nephrogenesis [134].

*Micro RNAs (miRNAs)* are small, non-coding RNAs that regulate gene expression at the post-transcriptional level. Over recent decades, miRNAs have proven to be stable in circulation, showing potential as biomarkers for the diagnosis and prognosis of several diseases, including GDM [135–138]. They regulate gene expression and play essential roles in biological processes such as development, differentiation, apoptosis, oncogenesis, metabolic homeostasis, and DNA methylation. Additionally, miRNAs are involved in maintaining glucose homeostasis and in the production and secretion of insulin [120, 121, 136, 139–141].

The role of micro RNAs in kidney development has been recently reviewed [138]. The expression of miRNAs is influenced by diet and plays a crucial role in determining nephron numbers by targeting various cellular processes [142]. Conditional ablation of the miRNA processing enzyme *Dicer1* leads to the premature depletion of NPCs, resulting in a radical reduction in nephron endowment and increased expression of the pro-apoptotic marker *Bim* [143, 144]. Interestingly, mice lacking both *Dicer1* and *Bim* show a higher nephron count than those lacking *Dicer1* alone, suggesting that miRNAs regulate NPC survival partly through control of *Bim* expression in the developing kidney [145].

The enzymes DICER and DROSHA are upregulated in the placenta of women GDM gestation [146]. However, despite upregulation of Dicer and Drosha only a portion of the expressed miRNAs were found to be altered in these placenta samples of women with GDM [147]. Since most miRNAs rely on DICER and DROSHA function for their biogenesis, this is unlikely to account for why only certain miRNAs are dysregulated in the placenta of individuals with GDM. An alternative hypothesis is that elevated glucose levels or other factors associated with the diabetic environment may directly influence miRNA expression in the placenta, the fetus and other maternal tissues, such as the developing kidneys. Therefore, GDM could impact miRNA expression differently in different organs or even in the mother vs. fetus.

One of the most studied miRNA species is the let-7 miRNA. Biogenesis of Let-7 is inhibited by the RNA-binding protein *Lin28b*. A recent study found that overexpressing *Lin28b* in Wilms tumor protein (Wt1)-expressing cells during kidney development or suppressing *Let-7* prolongs Six2 expression and increases glomerular counts. The *Let-7* family appears to regulate the timing of nephrogenic cessation and thus nephron number [148, 149]. However, this regulatory loop appears to be more complex since enhanced *Lin28b* expression in Wt1-positive cells results in additional fields of nephrogenic mesenchyme, while global *Let-*7 suppression extends nephrogenesis within the metanephric mesenchyme [148]. Interestingly, *Let-7* miRNAs are upregulated in response to maternal low-protein diet during gestation [142], suggesting an adaptive role in response to environmental conditions may be at play when nutrients are scarce.

## Epigenetic Modifications Induced by Parental Obesity and GDM

The initial studies on fetal programming focused on maternal undernutrition [60, 65]. However, animal and clinical studies soon showed that overnutrition can lead to epigenetically mediated alterations in various physiological homeostatic regulatory systems and is linked to increased cardiometabolic risk in offspring [4, 5, 150]. In fact, the correlation between birth weight and later-life risk of type 2 diabetes [150], and hypertension [151, 152] does not appear to be linear, but rather U-shaped. Thus, both low and high birth weights, above normal standards, seem to be major risk factors for metabolic diseases.

Genetics can influence body composition, but epigenetics also contributes to the phenotype significantly. The predicted genetic contribution to obesity and high body mass index is still unclear. Initial estimate attempts based on twin studies predicted genetic contribution to the obese body composition to range between 40 and 70% [153]. Later, more accurate estimates indicate that genetic contributions range from 30 to 40% as determining factors for high body mass [153, 154].

Recent evidence suggests a mechanistic link between altered epigenetic regulation of gene expression, parental obesity, and high-fat diets, highlighting their impact on the development of non-communicable diseases in offspring later in life [3, 152, 155–158]. Maternal obesity has been proposed to increase the risk of offspring developing conditions such as hypertension, CKD, and metabolic diseases in adulthood [3, 6, 8, 9, 22, 151]. Obesity is a multifactorial condition, and the specific mechanisms or factors that pose the greatest risk to embryonic development remain unclear. During pregnancy, some degree of insulin resistance and elevated circulating fatty acid and glucose levels are expected as part of normal physiological changes. However, 1 to 25% of all pregnant women develop gestational diabetes GDM, which, if left unregulated, exposes the fetus to prolonged periods of elevated hormones such as insulin and IGF, as well as increased circulating carbohydrates and fatty acids.

A study by Ruchat et al. (2013) found that GDM epigenetically modifies genes primarily involved in metabolic pathways. These changes may explain the increased risk of metabolic diseases in both mothers and offspring post-GDM [159].

Research distinguishing the effects of obesity combined with GDM from those of obesity alone on kidney development remains limited. Therefore, in this review, we focus on the impacts of GDM on the epigenetic regulation of kidney development. In the context of GDM, maternal hyperglycemia has been shown to induce specific epigenetic changes in the fetus, including DNA methylation, histone modifications, and the dysregulation of miRNAs, all of which can have lasting effects on kidney development and function [29, 39, 40, 43, 104].

The epigenetic modifications induced by GDM influence several molecular pathways essential to kidney development and function. One key pathway is the oxidative stress response, which is increased in GDM due to high glucose levels. Epigenetic changes, particularly the increased methylation of antioxidant response genes, reduce the kidney’s ability to counteract oxidative stress, leading to cellular damage during development [29, 40, 99, 104, 110, 160]. This chronic oxidative state can impair nephron formation and increase susceptibility to CKD (Fig. 2, left panel) [30].

DNA methylation, which typically (but not always) acts to repress gene expression, has been studied in GDM and kidney development [41, 126, 136, 161–163]. Research has shown that maternal hyperglycemia can alter DNA methylation at genes critical for kidney cell differentiation and function, potentially leading to abnormal development and susceptibility to disease [103, 105, 160, 164]. Some studies have reported hypermethylation of genes associated with kidney morphogenesis in the offspring of mothers with GDM, suggesting that these methylation changes could be an epigenetic mechanism through which GDM affects kidney development [158, 161, 165, 166].

The study by El Hajj et al. (2013) investigated the impact of GDM on the DNA methylation of the *Mest* gene in offspring. The researchers found that intrauterine exposure to GDM led to significant hypomethylation of the *Mest* gene in the offspring’s DNA. This epigenetic alteration was associated with increased expression of the *Mest* gene, which is implicated in adipose tissue development and metabolic regulation. The findings suggest that maternal GDM can induce epigenetic changes in the offspring, potentially predisposing them to metabolic disorders later in life [41].

The study by Ly et al. (2016) found that maternal folic acid supplementation affects DNA methylation and gene expression in rat offspring, depending on the timing during gestation and the specific organ. Early and late gestation supplementation resulted in different epigenetic and gene expression patterns, with organ-specific responses, particularly in the liver and brain. These changes influenced key developmental and metabolic genes, emphasizing the importance of timing and dosage of folic acid during pregnancy [126].

Haertle et al. (2017) identified specific DNA methylation changes in cord blood associated with GDM. These epigenetic alterations are linked to genes involved in metabolism and immune responses, suggesting a potential mechanism for the intergenerational transmission of metabolic risk [161].

Chen et al. (2017) demonstrated differential DNA methylation patterns in individuals exposed to maternal diabetes in utero. These changes were observed in genes associated with glucose metabolism and insulin signaling, highlighting the long-term epigenetic impacts of maternal diabetes on offspring [162].

Michalczyk et al. (2016) identified epigenetic markers, such as altered DNA methylation patterns, which predict the risk of conversion from GDM to type 2 diabetes. These markers are promising for early identification and prevention strategies [166].

Histone modifications, which regulate chromatin structure and gene accessibility, are also altered in response to GDM. Exposure to GDM can increase the acetylation of histones at specific genes that may be involved in kidney development and metabolic pathways, enhancing their expression and potentially disrupting the fine balance required for normal nephrogenesis [28, 159, 165–167].

Hepp et al. (2018) found that histone H3 lysine 9 acetylation is downregulated in placentas from GDM pregnancies. Calcitriol supplementation further reduced this acetylation, suggesting that both GDM and interventions like calcitriol influence placental epigenetic marks [165].

Certain miRNAs involved in kidney development are overexpressed or reduced in GDM-exposed offspring, which may contribute to kidney developmental anomalies and increase disease risk in adulthood [122, 135, 138, 168].

Borrero et al. (2023) performed a systematic review that analyzed miRNA expression profiles in GDM patients, identifying specific miRNAs associated with the condition. The findings suggest that these miRNAs are altered in GDM and could serve as biomarkers for early GDM detection and may offer insights into the disease’s pathophysiology [136].

Hohenstein and Hastie (2014) discussed the involvement of the LIN28 protein and miRNAs in kidney development and the formation of Wilms tumors. They highlight how dysregulation of miRNA pathways can contribute to tumorigenesis, emphasizing the importance of miRNAs in renal biology and cancer [137].

Cerqueira, Tayeb, and Ho (2022) reviewed the critical functions of miRNAs in kidney development and disease. The authors detailed how miRNAs regulate gene expression during nephrogenesis and their roles in various kidney pathologies, underscoring their potential as therapeutic targets for prevention and treatment of developmental problems of the kidney [138].

Wang et al. (2021) studied the circulating mRNAs in patients with GDM. The authors identified circulating miR-574-5p and miR-3135b as potential regulators of serum lipids and blood glucose levels in GDM patients [139].

Collectively, these studies underscore the significant roles of miRNAs in metabolic regulation, disease development, and their potential as biomarkers and therapeutic targets in conditions like GDM and kidney-related diseases.

GDM has been shown to epigenetically upregulate pro-inflammatory genes in fetal kidney tissue, establishing a pro-inflammatory environment. This inflammatory state can lead to fibrosis, a process in which kidney tissue is replaced with scar tissue, thereby impairing kidney function. Histone modifications in inflammatory genes may increase their expression, promoting inflammation and disrupting kidney development. Such inflammatory responses *in utero* may prime the kidneys for a heightened response to inflammatory insults in adulthood, increasing the risk of kidney diseases [94, 96, 169].

Dłuski et al. (2021) reviewed epigenetic changes linked to GDM, focusing on DNA methylation, histone modifications, and non-coding RNAs. The authors found that alterations in these epigenetic pathways affect the expression of metabolic and inflammatory genes, contributing to disease progression and potential long-term effects on the offspring [164].

GDM exposure can lead to abnormal nephron structure and function, contributing to renal impairment later in life. Understanding how GDM-induced epigenetic changes impact kidney development can provide insights into potential intervention strategies. Unfortunately, there is a need for appropriate animal models for GDM that can be used to understand the molecular mechanism of how GDM promotes epigenetic changes that impact kidney development and whether its impact is limited to a single generation or affects multiple generations.

## Potential for Therapeutic Interventions and Prevention

Addressing the long-term impacts of GDM on kidney development requires a focus on preventive and therapeutic strategies that target both maternal health and fetal development. Preventive strategies include optimizing maternal glycemic control through dietary modifications, physical activity, and insulin therapy if necessary. Studies have shown that maternal interventions to maintain normoglycemia can reduce the risk of developmental abnormalities in the kidneys of GDM-exposed offspring [39, 43].

Nutritional supplementation during pregnancy also holds promise. Nutrients such as folate and choline, which support DNA methylation and histone modification, could help buffer against the epigenetic impact of GDM on the fetal kidney. Dietary interventions that include antioxidants have shown potential in animal models, where they reduced oxidative stress markers in the offspring’s kidneys, suggesting a possible protective role against GDM-induced kidney alterations. However, there are limited studies on the efficacy of nutritional supplements for GDM, and the findings are not consistent. More large, high-quality studies are needed to determine the effectiveness of these supplements [77, 170–172].

Compounds that influence DNA methylation or histone modification, like folate or histone deacetylase inhibitors, may help normalize gene expression in fetal kidneys exposed to GDM. Finally, early postnatal interventions that include regular monitoring of renal health and metabolic conditions can help mitigate long-term consequences in individuals exposed to GDM *in utero*.

In summary, GDM is a major risk factor for the development of hypertension and CKD later in life. GDM dysregulates epigenetic mechanisms that are required for proper kidney development. The hyperglycemic environment causes depletion of nephron progenitors and impairs proper nephrogenesis, contributing to the oligonephropathy observed in the offspring of mothers who face gestational diabetes. It is of utmost clinical importance to develop new therapeutic strategies to prevent or treat GDM to ensure proper embryonic development.

## Conclusion

Understanding the developmental complications arising from gestational diabetes is critically important for improving long-term health outcomes, as it has significant implications for the onset of non-communicable diseases later in life. Embryonic kidneys are particularly vulnerable to the effects of gestational diabetes, with evidence from both animal models and human studies showing that offspring of mothers with gestational diabetes have an increased risk of developing hypertension and chronic kidney disease. Mechanistically, gestational diabetes can reprogram kidney development through epigenetic modifications, underscoring the need for targeted interventions that could mitigate these long-term risks. Table 1 lists the main papers showing the relationship between maternal health, epigenetic programming, and long-term metabolic and renal health risks in offspring.

**Table 1:** Research Summary on GDM and related topics. This table summarizes key research findings on gestational diabetes mellitus (GDM) and related topics, organized by primary subject. The “Primary Subject” column categorizes the studies into overarching themes such as maternal nutrition, epigenetics, and kidney development. The “References” column lists the corresponding studies, while the “Main Findings” column highlights the significant insights, including the role of maternal conditions, epigenetic modifications, microRNAs, and nutritional interventions in influencing offspring health outcomes

Primary subject	References	Main findings
Maternal Nutrition and GDM	[1, 2, 6, 25]	Maternal obesity and suboptimal nutrition during pregnancy are associated with adverse metabolic programming, increasing offspring’s risk for GDM, type 2 diabetes, and cardiovascular diseases.
Epigenetics in GDM	[41, 161, 162, 164, 165]	GDM induces DNA methylation changes and histone modifications in offspring, affecting metabolic pathways and increasing disease susceptibility. Histone H3K9 acetylation is downregulated in GDM placentas.
MicroRNAs in GDM	[136, 138, 139]	Specific miRNAs (e.g., miR-574-5p, miR-3135b) act as biomarkers and regulators of metabolic changes in GDM, impacting glucose and lipid metabolism.
Kidney Development	[45, 48, 50, 54, 127]	Epigenetic and molecular regulation, including histone modifications and signaling pathways, governs nephron progenitor maintenance and kidney organogenesis.
Long-Term Health Implications	[5, 30, 78, 86]	Intrauterine exposures, such as GDM or nutrient deficiencies, lead to nephron deficits, renal dysfunction, and increased risk of hypertension and kidney diseases in offspring.
Nutritional Interventions	[125, 126, 172]	Maternal folic acid supplementation and other dietary interventions during pregnancy influence DNA methylation, gene expression, and kidney development in offspring, potentially mitigating adverse effects of nutrient deficiencies or GDM.
Diabetes Programming	[14, 17, 169]	Exposure to maternal hyperglycemia during pregnancy predisposes offspring to metabolic and renal disorders, mediated by epigenetic programming.

## Key References

- Bashir M, Fagier Y, Ahmed B, C Konje J. An overview of diabetes mellitus in pregnant women with obesity. Best Pract Res Clin Obstet Gynaecol. 2024;93:102469.
  - This review elaborates on the current insights into the pathogenesis of endocrine disturbances linked to diabetes during pregnancy. It discusses the approach to screening, management strategies—including pre-pregnancy care—and the involvement of newer therapeutic agents in treatment.

- Wang H, Li N, Chivese T, Werfalli M, Sun H, Yuen L, et al. IDF Diabetes Atlas: Estimation of Global and Regional Gestational Diabetes Mellitus Prevalence for 2021 by International Association of Diabetes in Pregnancy Study Group’s Criteria. Diabetes Res Clin Pract. 2022;183:109050.
  - This review shows that the global prevalence of GDM varies widely across the globe, ranging from 1 to 25%. Factors such as diagnosis criteria and socio-economics are discussed.

- Aisa MC, Cappuccini B, Barbati A, Clerici G, Torlone E, Gerli S, et al. Renal Consequences of Gestational Diabetes Mellitus in Term Neonates: A Multidisciplinary Approach to the DOHaD Perspective in the Prevention and Early Recognition of Neonates of GDM Mothers at Risk of Hypertension and Chronic Renal Diseases in Later Life. J Clin Med. 2019 Mar 28;8(4):429.
  - This paper shows a direct measurement of the impact of GDM on kidney volume. The paper shows that neonates of mothers with strict normoglycemic control showed no differences in kidney volume compared to controls. In contrast, the descendants of mothers who lacked glycemic control had significantly lower renal volumes, which is a major factor in the development of CKD later in life.

- Lizárraga D, Gómez-Gil B, García-Gasca T, Ávalos-Soriano A, Casarini L, Salazar-Oroz A, et al. Gestational diabetes mellitus: genetic factors, epigenetic alterations, and microbial composition. Acta Diabetologica 2023 61:1.
  - This paper examines how environmental factors, through epigenetic mechanisms, influence disease risk. It highlights the association between microbial composition and GDM, suggesting that both epigenetic markers and microbiota could be passed to offspring, thereby increasing their likelihood of developing chronic degenerative conditions later in life.

- Hjort L, Novakovic B, Grunnet LG, Maple-Brown L, Damm P, Desoye G, et al. Diabetes in pregnancy and epigenetic mechanisms-how the first 9 months from conception might affect the child’s epigenome and later risk of disease. Lancet Diabetes Endocrinol. 2019;7:796–806.
  - This paper discusses that epigenetic changes established in utero may link the prenatal environment to future disease susceptibility. It indicates that identifying an epigenetic fingerprint associated with maternal diabetes could serve as a biomarker for early detection of at-risk offspring, enabling targeted monitoring and intervention.

- Parimi M, Nitsch D. A Systematic Review and Meta-Analysis of Diabetes During Pregnancy and Congenital Genitourinary Abnormalities. Kidney Int Rep. 2020;5:678–93.
  - This study indicates that 2.0–3.7% of cases of CAKUT in the United States, and up to 14% of CAKUT in some populations could be eliminated if GDM was prevented or eradicated.

## Data Availability

No datasets were generated or analysed during the current study.
